# Association of Fatigue and Stress With Gray Matter Volume

**DOI:** 10.3389/fnbeh.2018.00154

**Published:** 2018-07-24

**Authors:** Keisuke Kokubun, Kiyotaka Nemoto, Hiroki Oka, Hiroki Fukuda, Yoshinori Yamakawa, Yasuyoshi Watanabe

**Affiliations:** ^1^Office of Society-Academia Collaboration for Innovation, Kyoto University, Kyoto, Japan; ^2^Division of Clinical Medicine, Department of Neuropsychiatry, Faculty of Medicine, University of Tsukuba, Tsukuba, Japan; ^3^ImPACT Program of Council for Science, Council for Science, Technology and Innovation, Cabinet Office, Government of Japan, Chiyoda, Japan; ^4^Institute of Innovative Research, Tokyo Institute of Technology, Meguro, Japan; ^5^RIKEN Center for Life Science Technologies, Yokohama, Japan

**Keywords:** gray-matter brain healthcare quotient, magnetic resonance imaging data, profile of mood states, perceived stress scale, chalder fatigue scale, fatigue, stress

## Abstract

Stress is associated with a greater risk for various health problems including reduced gray matter volume (GMV) and density in a number of brain regions. Previous studies show that neuroimaging could be a means to objectively evaluate stress. However, to date, no definite neuroimaging-derived measures are available to detect stress. In this research we used the gray-matter brain healthcare quotient (GM-BHQ), an MRI-based quotient for monitoring brain health based on GMV, as an objective scale to measure the association of stress with the whole brain. We recruited 63 healthy adults to acquire structural T1-weighted images and stress levels evaluated using three representative stress scales: the Profile of Mood States (POMS), Perceived Stress Scale (PSS) and Chalder Fatigue Scale (CFS). We found that the GM-BHQ was sensitive to fatigue and the interaction between fatigue and stress.

## Introduction

A number of studies of chronic stress have shown that stress is associated with a greater risk for various health and health-related problems such as decreased work ability, increased caloric intake followed by obesity, insomnia, dementia, and others (Cuadros et al., 2012; Greenberg et al., 2014; da Silva et al., 2016). Stress can lead to reduced labor productivity and increased medical expenses, which could weaken the financial foundation of a nation. In this context, utilizing an objective method for evaluating the influences of stress or fatigue on people's lives would provide very useful information upon which to base lifestyle changes to minimize that stress.

In the area of neuroscience, it has already been reported that stress is associated with reduced gray matter volume (GMV) and density in a number of brain regions (Li et al., 2014), including the anterior cingulate cortex (ACC), medial-orbital frontal cortex, hippocampus and insula (Corbo et al., 2005; Thomaes et al., 2010; Woon et al., 2010). Though subjects of most of these studies are patients with specific diagnoses, post-traumatic stress disorder, there are a few studies which explored the influence of stress on the brains of healthy people. Ansell et al. (2012) found a reduction of the GMV of the ACC in healthy people who suffered daily stress, which shows that stress influences the brains even of healthy people. And also in the stress- and fatigue-related severe diseases, such as Myalgic Encephalomyelitis/Chronic Fatigue Syndrome, one of co-authors' group found the reduced GMV in the prefrontal cortices and its close relationship with the severity of fatigue extent (Okada et al., 2004).

Previous studies show the possibility that neuroimaging could be a means to objectively evaluate stress. However, to date, no definite neuroimaging-derived measures are available to assess stress. Recently, efforts have been made to derive various measures from neuroimaging data; researchers engaged with UK Biobank call these “image-derived phenotypes” (IDP) and describe that they are intended to be useful for individuals who are not imaging experts (Miller et al., 2016). We have also developed a measure, called the “gray-matter brain healthcare quotient,” which is an average of standardized gray matter measures for 116 brain regions based on the AAL atlas (Tzourio-Mazoyer et al., 2002), and we found that the GM-BHQ is inversely correlated with age as well as obesity (Nemoto et al., 2017).

Since the GM-BHQ is a measure which reflects gray matter volume, we hypothesized that it might be associated with stress and/or fatigue. Therefore, in this study we investigated the relations between the GM-BHQ of healthy participants and three representative stress scales: The Profile of Mood States (POMS: McNair et al., 1971), Perceived Stress Scale (PSS: Cohen and Williamson, 1998), and Chalder Fatigue Scale (CFS: Chalder et al., 1993).

## Materials and methods

### Subjects

Eighty-five healthy participants (40 females and 45 males) were recruited in Kyoto, Japan. Potential participants who had medical histories of neurological, psychiatric, or medical conditions that could affect the central nervous system were excluded from recruitment. After recruitment, we administered the Center for Epidemiologic Studies Depression (CES-D) scale (Radloff, 1977) to screen for depression. The CES-D scores of 22 participants were higher than 16 points, so we excluded them because of the potential that they were suffering from depression. Thus, the analysis included 63 participants (34 females and 29 males), aged 40-69 (mean (M) ± standard deviation (SD): 53.2 ± 8.3 years). This study was approved by the ethics committees of Kyoto University (approval number 27-P-13) and performed in accordance with the guidelines and regulations of the institute. All participants gave written informed consent prior to participation, and participant anonymity has been preserved.

### Stress factors

We employed three sets of questionnaires, POMS, PSS, and CFS, to evaluate the stress and fatigue of participants. The Profile of Mood States (POMS) scale is a 65-item questionnaire measuring six mood states: 9 items for tension-anxiety (tension), 15 items for depression-dejection (depression), 12 items for anger-hostility (anger), 7 items for fatigue-inertia (fatigue), 8 items for vigor-activity (vigor), and 7 items for confusion-bewilderment (confusion) on a five-point Likert scale from 0 (not at all) to 4 (extreme). The scale was developed by McNair et al. (1971) and higher scores reflect mood decrements, with the exception of the vigor subscale, where higher scores reflect improved mood.

The Perceived Stress Scale (PSS), developed by Cohen and Williamson (1998), is a measurement of the degree to which situations in life are appraised as stressful on a five-point Likert scale from 0 (never) to 4 (very often) and the most widely used psychological instrument for measuring the perception of emotional stress.

The Chalder Fatigue Scale (CFS) is also one of the most frequently employed scales. It was developed to measure the severity of fatigue on a four-point scale from 0 (less than usual) to 4 (much more than usual) by Chalder et al. (1993). The 11 questions of the CFS are divided into two dimensions: 7-item physical fatigue and 4-item mental fatigue.

To the best of our knowledge, POMS has typically been used to measure mood or temporal psychological state while PSS and CFS have been used for the measurement of more chronic or long-term stress and fatigue. Therefore, we were able to consider what kind of stress or fatigue most affected the GM-BHQ by comparing the analytical results of these three questionnaires.

### MRI data acquisition

All magnetic resonance imaging (MRI) data were collected using a 3-T Siemens scanner (Verio, Siemens Medical Solutions, Erlangen, Germany or MAGNETOM Prisma, Siemens, Munich, Germany) with a 32-channel head array coil. A high-resolution structural image was acquired using a three-dimensional (3D) T1-weighted magnetization-prepared rapid-acquisition gradient echo (MP-RAGE) pulse sequence. The parameters were as follows: repetition time (TR), 1,900 ms; echo time (TE), 2.52 ms; inversion time (TI), 900 ms; flip angle, 9°; matrix size, 256 × 256; field of view (FOV), 256 mm; and slice thickness, 1 mm.

### MRI data analysis

GM-BHQ was calculated according to our previous study (Nemoto et al., 2017). In summary, gray matter images were segmented from T1-weighted images using Statistical Parametric Mapping 12 (SPM12; Wellcome Trust Center for Neuroimaging, London, UK) running on MATLAB R2015b (Mathworks Inc., Sherborn, MA, USA), followed by spatial normalization using diffeomorphic anatomical registration through an exponentiated lie algebra (DARTEL) algorithm (Ashburner, 2007) and modulation to preserve the GM volume. All normalized, segmented, and modulated images were smoothed with an 8-mm full width at half-maximum (FWHM) Gaussian kernel. Additionally, intracranial volume (ICV) was calculated by summing the GM, white matter, and cerebrospinal fluid images for each subject. Proportional GM images were generated by dividing smoothed GM images by ICV to control for differences in whole-brain volume across participants. Using these proportional GM images, images for the mean and standard deviation (SD) across participants were generated. Then, we calculated the GM-BHQ using the following formula: 100 + 15 × (individual proportional GM - mean) / SD. Regional GM quotients were then extracted using an automated anatomical labeling (AAL) atlas (Tzourio-Mazoyer et al., 2002) and averaged across regions to produce participant-specific GM-BHQ.

### Statistical analysis

In order to investigate the correlation between the GM-BHQ and various variables, we employed hierarchical regression analysis. We entered the control variables of age and sex in Step 1 and the main effects of sequential stress-related variables in Step 2. In Step 3, we entered interaction terms of stress variables with main variables of significant association from Step 2. Independent variables were selected using the stepwise method because there were many stress-related variables. We added these respective variables to the models based on the hypothesis that stress and its interaction terms are closely related to GM-BHQ after adjusting for age and sex. The significance level was determined at p < 0.05. All statistical analyses were performed using IBM SPSS Statistics Version 20 (IBM Corp., Armonk, NY, USA). Data used for the analysis is provided in Table S1.

## Results

Descriptive statistics of subjects and correlation coefficients between psychological scales are shown in Table 1. The internal consistencies (Cronbach's alpha) for the six components of POMS (tension, depression, anger, vigor, fatigue and confusion) were significant (α = 0.788, α = 0.930, α = 0.918, α = 0.912, α = 0.912 and α = 0.778, respectively) as were the PSS (α = 0.733) and the two components of the CFS (physical, mental; α = 0.875 and α = 0.784, respectively). GM-BHQ correlated with age (*r* = −0.543, *p* < 0.001) and sex (*r* = 0.529, *p* < 0.001). Overall, in many cases, stress-related variables were significantly correlated with one another. However, they were not significantly correlated with GM-BHQ, age or sex. GM-BHQ was correlated with age, sex and, marginally, POMS (fatigue; r = −0.222, *p* < 0.10).

**Table 1:** Descriptive statistics and correlations.

**Variables**	**Mean**	**SD**	**α**	**1**	**2**	**3**	**4**	**5**	**6**	**7**	**8**	**9**	**10**	**11**	**12**
1 GM-BHQ	96.463	7.100	–												
2 Age (male = 1, female = 2)	53.159	8.291	–	−0.543^***^											
3 Sex	1.540	0.502	–	0.529^***^	−0.125										
4 POMS (tension)	7.968	4.048	0.788	−0.098	−0.102	−0.126									
5 POMS (depression)	5.397	4.467	0.930	−0.140	−0.052	−0.126	0.635^***^								
6 POMS (anger)	7.143	5.524	0.918	−0.203	0.019	−0.208	0.610^***^	0.612^***^							
7 POMS (vigor)	16.079	6.126	0.912	0.046	−0.025	−0.150	0.077	−0.074	0.085						
8 POMS (fatigue)	6.413	4.553	0.912	−0.222^†^	0.004	−0.078	0.626^***^	0.553^***^	0.547^***^	−0.087					
9 POMS (confusion)	6.603	3.372	0.778	−0.191	−0.072	−0.157	0.531^***^	0.570^***^	0.442^***^	−0.294^*^	0.522^***^				
10 PSS	21.381	4.484	0.733	−0.017	−0.160	0.058	−0.070	0.263*	0.086	−0.478^***^	−0.096	0.319^*^			
11 CFS (physical)	5.365	3.409	0.875	−0.087	−0.025	0.156	0.216	0.233	0.258	−0.291	0.416^**^	0.247	0.257^*^		
12 CFS (mental)	2.889	1.627	0.784	−0.080	−0.066	0.035	0.313	0.414^**^	0.294^*^	−0.449^***^	0.509^***^	0.544^***^	0.311^*^	0.682^***^	

n = 63.

† p < 0.10.

* p < 0.05.

** p < 0.01.

*** *p < 0.001*.

Table 2 shows the results of regression analyses. In Step 1, both age (*R* = 0.715, *b* = −0.484, *p* < 0.001) and sex (*R* = 0.715, *b* = 0.469, *p* < 0.001) were significant, which means that GM-BHQ scores tended to be lower in male and elderly than female and younger participants. In Step 2, POMS (fatigue) was significantly associated with a lower GM-BHQ (*R* = 0.738, *b* = −0.185. *p* = 0.040). In Step 3, two interaction variables, POMS (fatigue) ^*^ PSS (*R* = 0.760, *b* = −0.258, *p* = 0.004) and POMS (fatigue) ^*^ Chalder (physical; *R* = 0.749, *b* = −0.224, *p* = 0.012), were significantly associated with lower GM-BHQ. However, in the same Step 3, POMS (fatigue) was not selected as a significant variable. Furthermore, none of the variables, except those which appear in the table, were significantly correlated with GM-BHQ.

**Table 2:** Multiple regression analysis of stress factors on GM-BHQ.

	**GM-BHQ**							
	**Step 1**		**Step 2**		**Step 3**			
	**β^a^^,^^b^**	***p*-value**	**β^a^^,^^b^**	***p*-value**	**β^a^^,^^b^**	***p*-value**	**β^a^^,^^b^**	***p*-value**
**CONTROL VARIABLES**								
Age	−0.484	<0.001^***^	−0.485	<0.001^***^	−0.500	<0.001^***^	−0.487	<0.001^***^
Sex (male = 1, female = 2)	0.469	<0.001^***^	0.454	<0.001^***^	0.446	<0.001^***^	0.481	<0.001^***^
**MAIN VARIABLES**								
POMS (fatigue)			−0.185	0.040^*^				
**INTERACTION VARIABLES**								
POMS (fatigue) * PSS					−0.258	0.004^**^		
POMS (fatigue) * CFS (physical)							−0.224	0.012^*^
*R*	0.715	<0.001^***^	0.738	<0.001^***^	0.760	<0.001^***^	0.749	<0.001^***^
*R*^2^	0.511		0.545		0.577		0.561	

n = 63.

* p < 0.05.

** p < 0.01.

*** p < 0.001.

a Standardized regression coefficient.

b Independent variables were selected using the stepwise method.

The summary of the results is as follows. First, fatigue, as measured by POMS, has a negative correlation with GM-BHQ after adjusting for age and sex. This means, in short, more fatigue correlates with a lower GM-BHQ. Second, fatigue, as measured by POMS, may negatively correlate with GM-BHQ more significantly when it interacts with other stress. That is, the more people experience fatigue together with stress or chronic physical fatigue, the lower their GM-BHQ tends to be.

## Discussion

Stress is associated with a greater risk for various health problems including reduced gray matter volume (GMV) and density in a number of brain regions (Li et al., 2014). Previous studies have shown that neuroimaging could be a means to objectively evaluate stress. However, to date, no definite neuroimaging-derived measures are available for stress. In this research we used the gray-matter brain health care quotient (GM-BHQ), an MRI-based quotient developed in our previous study (Nemoto et al., 2017) for monitoring brain health based on GMV, as an objective scale to measure the effect of stress on the whole brain.

We employed three sets of psychological scales, Profile of Mood States (POMS), Perceived Stress Scale (PSS), and Chalder Fatigue Scale (CFS) to evaluate the stress and fatigue of participants. Among them, POMS is a well-validated tool commonly used in medical/clinical research. In recent studies, the scale has been used to measure mood states of patients who, for example, underwent epilepsy treatment (Szaflarski et al., 2017) or thyroid removal surgery (Lombardi et al., 2017). In the area of neuroscience, Dong et al. (2017) examined the correlation between mood states and functional connectivity (FC) among default mode network (DMN) regions and found that the FC allowed the differentiation of participants with Internet gaming disorder (IGD) from healthy controls (HC).

On the other hand, in recent research, PSS has been used to study the relations between stress and body pain (White et al., 2014) or stress and allergy attacks (Patterson et al., 2014). Furthermore, there is a study which investigated the effect of social capital (i.e., constructing trust with the peers) on the lessening of stress (Chen et al., 2015). In the area of neuroscience, Aggarwal et al. (2014) revealed that increasing levels of stress are associated with accelerated declines in cognitive function in adults 65 years and older. Furthermore, Evans et al. (2014) showed that childhood poverty was associated with reduced DMN connectivity and in turn was associated with higher cortisol levels in anticipation of social stress. Another scale, CFS, has been used to assess the severity of fatigue not only of patients with chronic fatigue syndrome (Chalder et al., 2015), cancer (Goedendorp et al., 2016), multiple sclerosis (Chilcot et al., 2016), and others, but also of the general population (Jing et al., 2016).

By analyzing the relations between healthy participants' GM-BHQ and the results of these three representative stress scales (POMS, PSS and CFS), we found that GM-BHQ may be low in a person suffering from fatigue or a combination of fatigue and stress. In other words, managing fatigue and stress in our daily lives could prevent decreases in GM-BHQ, which is to say brain atrophy. The GM-BHQ, as an objective measure of the association of stress or fatigue with the brain, might provide useful information upon which to base lifestyle changes aimed at reducing stress and fatigue.

It has been said that there are a number of mechanisms through which stress may affect GMV, including loss of neurons, decreased dendritic branching, spine density, and decreased neurogenesis (Arnsten, 2009; Lupien et al., 2009; Ansell et al., 2012). Previous research indicates significant loss in the anterior cingulated cortex (ACC) and hippocampus following post-traumatic stress disorder (PTSD) arising from childhood abuse or after experiencing trauma without the development of subsequent PTSD (Rogers et al., 2009; Dannlowski et al., 2012). In line with this, it is indicated in another research that fatigue in multiple sclerosis is related to significant resting-state functional connectivity reorganization in the principal resting-state networks, including an antero-posterior reorganization of the default-mode network and a remodeling of the sensorimotor network (Bisecco et al., 2017). As for cell level discussion, it is found that mitochondrial dysfunction is associated with chronic fatigue symptoms and reduced hippocampal and subcortical GMV (Kallianpur et al., 2016; Torrell et al., 2017).

Subjects experiencing stressful life events and cumulative life adversities, with no psychiatric diagnosis, also show GMV loss in the ACC (Ganzel et al., 2008; Papagni et al., 2011; Ansell et al., 2012). Thus, changes in GMV in a particular area, such as the ACC, the hippocampus, etc., may be associated not only with stress-related psychiatric disorders, but also with recent adverse life events (Kassem et al., 2013). Our results are in line with the findings of previous research and at the same time offer new knowledge that the condition of the whole brain, as measured by the GM-BHQ, is related to fatigue and stress in healthy people. The findings of this research are meaningful as it is known that reduced GMV is associated with a greater risk for health problems; for instance, Altamura et al. (2017) found that acute psychosis patients with a lower GMV had significantly poorer improvement in clinical variables after paliperidone administration compared to patients with a higher GMV, suggesting that GMV predicts drug response, with a higher GMV potentially supporting a favorable prognosis.

Let us consider why fatigue, as measured with POMS, has a negative correlation with the GM-BHQ while other factors do not. This question comprises two sub-questions: one asks about the difference between POMS and other measures (PSS and CFS), and the other asks about the difference between POMS (fatigue) and the other five POMS mood states (tension, depression, etc.). One possible response to the first sub-question may be that GM-BHQ is more sensitive to the instantaneous fatigue measured by POMS than to other kinds of stresses or to more chronic fatigue, perhaps due to brain plasticity. For instance, Kühn et al. (2014) found significant gray matter increase in the right hippocampal formation (HC), right dorsolateral prefrontal cortex (DLPFC) and bilateral cerebellum when participants trained with a commercial video game for 2 months for at least 30 min per day. Supposing such changeability in the brain, scales other than those which measure current mood states may not be appropriate. However, if a momentarily perceived sensation is not fatigue, but rather some other feeling, it may be too weak to be associated with the brain, even temporarily. This leads us to the second sub-question. Of course, the current results do not indicate that chronic stress or fatigue are meaningless. Rather GM-BHQ is most likely to be low when people experience additional temporal fatigue concurrently with chronic fatigue or chronic stress, as shown above.

There are some limitations to this study. First, the small sample size employed may have influenced the generalizability of our results. Second, other emotional scales unused in this research may have improved the interpretability of the data. Third, information on other factors that might influence brain volume such as alcohol/smoke habits of the subjects, sleep disturbances, anxiety may have increased robustness of the regression model. In summary, future studies are recommended to explore the relationship between GM-BHQ and more various emotional states, as measured through questionnaires, using larger sample sizes and more health-related control variables to clarify in greater detail the mechanisms which connect them.

## Author contributions

KN and YY: conceptulization; HO: data curation; KK: formal analysis; YY: funding acquisition, Investigation; KN: methodology; HF: project administration; YY: supervision; KK: writing–original draft. YW and KN writing–review and editing.

### Conflict of interest statement

The authors declare that the research was conducted in the absence of any commercial or financial relationships that could be construed as a potential conflict of interest.

## Supplementary material

Table S1 Data used for regression analyses of stress factors on GM-BHQ.
